# A Divide-and-Conquer Bat Algorithm with Direction of Mean Best Position for Optimization of Cutting Parameters in CNC Turnings

**DOI:** 10.1155/2022/4719266

**Published:** 2022-02-23

**Authors:** Xingwang Huang, Zongbao He, Yong Chen, Shutong Xie

**Affiliations:** ^1^School of Computer Engineering, Jimei University, Xiamen 361021, China; ^2^Digital Fujian Big Data Modeling and Intelligent Computing Institute, Jimei University, Xiamen 361021, China

## Abstract

Optimization of machining parameters is an important problem in the modern manufacturing world due to production efficiency and economics. This problem is well known to be complex and is regarded as a strongly nondeterministic polynomial (NP)-hard problem. To reduce the production cost of work-pieces in computer numerical control (CNC) machining, a novel optimization algorithm based on a combination of the bat algorithm and a divide-and-conquer strategy is proposed. First, the basic bat algorithm (BA) is modified with the aim to avoid finding the local optimal solution. In addition, a Gaussian quantum bat algorithm with direction of mean best position is developed. Second, in order to reduce the complexity of the optimization problem, the whole optimization problem is divided into several subproblems by using a divide-and-conquer strategy according to the characteristic of multipass turning operations. Finally, under a large number of machining constraints, the cutting parameters of the two stages of roughing and finishing are simultaneously optimized. Simulation results show that the proposed algorithm can find better combinations of the machining parameters than other algorithms proposed previously to further reduce the production cost. In addition, the outcome of our work presents a novel way to solve the complex optimization problem of machining parameters with a combination of traditional mathematical methods and swarm intelligence algorithms.

## 1. Introduction

In the manufacturing field, computer numerical control (CNC) machining refers to the computerized digital control of automated machine tools used to process rough material into semifinished or finished parts; it is one of the most common technologies. The main purpose of CNC machining is to save machining costs and improve machining efficiency and machining quality. Machining costs can be saved by selecting reasonable machining parameters, which introduces an optimization problem, i.e., selecting the optimal machining parameters to achieve the goal of reducing machining costs under the given machining constraints. Earlier research on the optimization of machining parameters mainly used traditional mathematical processing methods such as dynamic programming, sequential unconstrained minimization technique (SUMT), and linear or nonlinear programming. However, in general, the optimization problems of machining parameters are nonlinear and complicated problems with multiple constraints. Therefore, it is difficult to obtain satisfactory optimization solutions using traditional methods [1, 2]. In recent years, many scholars have applied swarm intelligence algorithms to the optimization problems of machining parameters in the field of computer integrated manufacturing. By using swarm intelligence algorithms to search for approximate optimal solutions of the problem, some research results have been achieved [3–16].

However, most previous studies were devoted to combining swarm intelligence algorithms with various local improvement algorithms [3–11, 17] (e.g., population diversification, local greedy search, and the use of heuristics as local search) in the hope of obtaining better results. However, because they did not fully consider the characteristics of the turning problem with multiple machining processes, the results obtained by the algorithms were similar, and it was difficult to significantly reduce the machining cost. To address this bottleneck, this paper proposes a novel optimization algorithm by fully considering the characteristics of the turning problem while effectively exploiting the global optimization performance of the swarm intelligence algorithm. By combining the improved bat algorithm with the divide-and-conquer strategy, the performance of the optimization algorithm is substantially improved. The final optimization algorithm is able to find better results.

The rest of this paper is organized as follows.  Section 2 represents the related works, especially the intelligent algorithms for optimization problems in CNC turning.  Section 3 describes the mathematical model for the optimization of machining parameters in CNC turnings.  Section 4 first introduces the bat algorithm, then proposes the Gaussian quantum bat algorithm with direction of mean best position (GQMBA), and finally elaborates on the idea of the combination of GQMBA and the divide-and-conquer strategy for solving the machining parameter optimization problems. In Section 5, simulation experiments are conducted, and different algorithms are compared. Finally, the concluding comments and some future research directions are presented in the last section.

## 2. Related Works

Optimization of turning parameters is an important issue in the manufacturing field. Early studies used traditional mathematical methods to find optimized machining parameters. Metaheuristic algorithms are also used to solve optimization problems of machining parameters. Chen and Tsai first proposed a mathematical model for the optimization problem of machining parameters in turnings and then combined Hooke-Jeeves pattern search (PS) into the simulated annealing (SA) algorithm to form a hybrid optimization algorithm (SA/PS) to solve the optimization problem [3]. Onwubolu and Kumalo [4] proposed a genetic algorithm (GA) to optimize the machining parameters in turnings but did not consider the constraint that the number of rough passes must be an integer. Chen and Chen [5] pointed out this shortcoming in the research of Onwubolu and Kumalo [4]. However, the optimization results obtained by the GA corrected by Chen and Chen were not better than those obtained by SA/PS. Additionally, based on a GA, Sankar [6] used a modified genetic algorithm (MGA) to search for optimized cutting parameters in turnings. The improved MGA used a specific crossover operator and three different mutation operators to enhance the diversity of the population and prevent the algorithm from converging to a local optimal solution. In addition to SA and GA, some studies applied other intelligent algorithms to the optimization problem of machining parameters. Vijayakumar [7], Wang [8], and Xie and Guo [16] developed new heuristic algorithms to overcome optimization problems based on the ant colony optimization (ACO) algorithm. In addition, the particle swarm optimization algorithm (PSO) is also one of the most widely used swarm intelligence methods [18]. Srinivas et al. proposed a PSO algorithm where the inertia coefficient decreased linearly with every iteration to solve the cutting parameter optimization problem [9]. Yildiz [10] and Costa et al. [12] also contributed different solutions based on the PSO algorithm to the problem. After comprehensive analysis of the previous research methods and results, Raja and Baskar [11] applied three optimization algorithms (SA, GA, and PSO) to three different machining parameter optimization models (single-pass turning, multipass turning, and surface grinding) to conduct experiments and compare the results of various types of intelligent optimization algorithms to the machining parameter optimization problem. The results showed that the optimization effect and computational efficiency of PSO are better than those of SA and GA. Scatter search (SS) is one of the optimization algorithms developed in the field of metaheuristics. Chen [19] focused on the application of the scatter search method in solving the optimization problem in turnings. By comparing it with other algorithms, the experimental results showed that the SS obtained superior machining parameters than some of the metaheuristic methods.

In recent years, in addition to the abovementioned classical intelligent algorithms, some new swarm intelligent algorithms have been proposed by researchers. Xu et al. proposed an improved flower pollination algorithm (FPA) and compared the obtained results with those of related studies [20]. Mellal and Williams used the cuckoo optimization algorithm (COA), one of the advanced bioinspired optimization algorithms, to minimize the unit production cost [21]. The experimental results showed that the COA algorithm is very competitive compared with other algorithms. Due to the successful application of swarm intelligence algorithms for optimization problems, Sofuoğlu et al. used three heuristic algorithms, GA, PSO and COA, to solve three different problems, which were more efficient and effective than other algorithms [22]. Similarly, Yildiz developed a new hybrid optimization algorithm to minimize the production cost by adding the Taguchi method that actively acted on the differential evolution algorithm to form a hybrid Taguchi-differential evolution algorithm (HRDE) [23]. The results showed that the hybrid algorithm was more effective than evolutionary algorithms presented in many related studies. In another work by Yildiz, he proposed a similar hybrid optimization method to determine the optimal machining parameters [24]. This method combined the differential evolution algorithm and receptor editing algorithm (DERE). The goal of the mathematical model was to determine the optimal machining parameters to reduce the unit production cost. The method has been experimentally proven to be an effective technique for optimizing machining parameters. Furthermore, in 2013, Yildiz proposed a parameter optimization method based on the artificial bee colony (ABC) algorithm [25] and a hybrid robust teaching-learning-based optimization algorithm (HRTLBO) based on the combination of guided learning optimization and the Taguchi method [26]. Compared with other methods, these proposed algorithms perform well, and better solutions can be found with them. Belloufi et al. provided specific application examples to illustrate the effectiveness of the proposed firefly algorithm (FA) for parameter optimization in multipass turnings [27].

## 3. Mathematical Model for Optimizing Machining Parameters in CNC Turnings

To optimize the machining parameters in multipass turnings, the mathematical model proposed in the literature [3, 20] takes a large number of actual machining constraints into account and is closer to real-world machining. Since the model has been cited in many studies, the optimization model is used in this paper. The cutting parameters to be optimized include rough cutting speed *V*_*r*_, rough feeding rate *f*_*r*_, rough depth of cut *d*_*r*_, the number of rough cuts *n*, finish cutting speed *V*_*s*_, finish feeding rate *f*_*s*_, and finish depth of cut *d*_*s*_. The unit production cost (UC) consists of the following four components:

1. Machining cost during real cutting time *C*_*M*_
2. Machine idle cost for setup operations and tool idling motion *C*_*I*_
3. Cost of tool replacement *C*_*R*_
4. Tool cost *C*_*T*_

Thus, the UC can be expressed as follows:

(1) $$\begin{array}{c} \;UC=\;C_{M}+C_{I}+C_{R}+C_{T},\\ \;=\left[\frac{\pi \;DL}{1000V_{r}f_{r}}\left(\frac{d_{t}-d_{s}}{d_{r}}\right)+\frac{\pi \;DL}{1000V_{s}f_{s}}\right]k_{0},\\ \;+\left[t_{c}+\left(h_{1}L+h_{2}\right)\left(\frac{d_{t}-d_{s}}{d_{r}}+1\right)\right]k_{0},\\ \;+\left[\frac{\pi \;DL}{1000V_{r}f_{r}}\left(\frac{d_{t}-d_{s}}{d_{r}}\right)+\frac{\pi \;DL}{1000V_{s}f_{s}}\right]\frac{t_{e}}{T_{p}}k_{0},\\ \;+\left[\frac{\pi \;DL}{1000V_{r}f_{r}}\left(\frac{d_{t}-d_{s}}{d_{r}}\right)+\frac{\pi \;DL}{1000V_{s}f_{s}}\right]\frac{k_{t}}{T_{p}}. \end{array}$$

where *k*_*0*_ is the sum of worker cost and management cost per unit time ($/min). *D* and *L* are the diameter and length of the work-piece (mm), respectively. *d*_*t*_ is the depth of material to be removed (mm). *h*_*1*_, *h*_*2*_ are the constants related to tool idle time and tool-in/out time, respectively. *t*_*c*_, *t*_*e*_ are the preparation time for loading and unloading time (min) and time required to exchange a tool (min), respectively. *T*_*p*_ is the tool life (min). *k*_*t*_ is the cutting edge cost ($/edge).

The number of the rough cut is as follows:

(2) $$\begin{array}{c} n=\;\frac{d_{t}-d_{s}}{d_{r}}\;,\;n\in z^{+}. \end{array}$$

The objective of the model is to minimize the UC (*V*_*r*_, *V*_*s*_, *f*_*r*_, *f*_*s*_, *d*_*r*_, *d*_*s*_, *n*) under many machining constraints on rough and finish turnings. The constraints are summarized as follows [3–8]:

1. The upper and lower constraints of *V*_*r*_, *V*_*s*_, *f*_*r*_, *f*_*s*_, *d*_*r*_, and *d*_*s*_
2. Tool life constraints
3. Cutting force, cutting power, and surface roughness constraint
4. Stable cutting region constraint; chip-tool interface temperature constraint
5. Constraints on the interconnection between roughing and finishing parameters

## 4. Optimization Algorithm Based on the Bat Algorithm and the Divide-and-Conquer Strategy

### 4.1. Overview of the Bat Algorithm

The bat algorithm (BA) [28] is a swarm intelligence search algorithm proposed to simulate the echolocation mechanism of bats when foraging. It achieves the localization of search targets by continuously adjusting the frequency and loudness of sound waves. It uses frequency tuning to increase the population diversity and uses automatic scaling to maintain a balance between global and local searches. The frequency, velocity, and position of the *i-*th bat in the bat population are represented as follows:

(3) $$\begin{array}{c} f_{i}=f_{\text{min}}+\left(f_{\text{max}}-f_{\text{min}}\right)\beta ,\\ v_{i}^{t}=v_{i}^{t-1}+\left(v_{i}^{t}-gb^{t}\right)f_{i}\;,\\ x_{i}^{t}=x_{i}^{t-1}+v_{i}^{t}. \end{array}$$

Here, *f*_*i*_ denotes the ultrasound frequency emitted by the *i*-th bat. *f*_max_ and *f*_min_ denote the upper and lower bounds of ultrasound frequency, respectively. *β* denotes a random number generated by a uniform distribution within the range of [0,1]. *v*_*t*_^*t*^ and *v*_*t*_^*t*−1^ denote the velocity of the *i*-th bat during the *t*-th and (*t*−1)-th iterations, respectively. *x*_*i*_^*t*^ and *x*_*i*_^*t−1*^ denote the position value of the *i*-th bat during the *t*-th and (*t*−1)-th iterations, respectively. The above expressions ensure the global search capability of the algorithm.

In the local search phase, BA uses a random walk strategy to generate feasible solutions at candidate locations. This strategy can be given by the following equation:

(4) $$\begin{array}{c} x_{\text{new}}=x_{\text{old}}+\varepsilon {\bar{A}}^{t}. \end{array}$$

where *ε* is the random number generated by the uniform distribution on the range [−1, 1] that determines the direction of the new candidate feasible solution and ‾*A*^*t*^ denotes the average acoustic loudness of all bats in the *t-*th iteration.

During the foraging process, as the bat approaches the foraging target, the bat will gradually adjust the loudness *A* and emission rate *r* of the ultrasound, making the loudness gradually decrease and the emission rate gradually increase to achieve more accurate positioning. The process is shown in the following equations:

(5) $$\begin{array}{c} A_{i}^{t}=\alpha A_{i}^{t-1}. \end{array}$$

(6) $$\begin{array}{c} r_{i}^{t}=r_{i}^{0}\left[1-\text{exp}\left(-\gamma t\right)\right]. \end{array}$$

In formula (6), *r*_*i*_^0^ denotes the initial pulse emission rate of the *i*-th bat, and both *α* and *γ* are constants between (0, 1).

### 4.2. Overview of the Quantum-Behaved Bat Algorithm with Mean Best Position Directed

Due to the lack of population diversity in the original BA, there is the problem of falling into local optima during the search. By analyzing the flight trajectory of bats, Zhu et al. proposed the quantum-behaved bat algorithm with mean best position directed (QMBA) [29]. The quantum computing mutation operator introduced by the algorithm can enhance population diversity and avoid premature convergence. At the same time, its average optimal position introduced in the local search phase can improve the convergence speed in the later stage of the search. The QMBA still retains the main body of the BA, which controls the global search and local search based on the ultrasonic loudness and the sending rate. The difference lies in its improved position update formula and local search strategy.

The position update formula in QMBA introduces a mechanism for adaptively adjusting the step size according to the distance. Its strategy for updating the position is expressed as follows:

(7) $$\begin{array}{c} x_{i\;d}^{t}=\left\{\begin{array}{c} x_{i\;d}^{t-1}+\left(gb_{d}-x_{i\;d}^{t-1}\right)\eta ,\;\delta_{d}>TH\\ x_{i\;d}^{t-1}+\epsilon ,\delta_{d}\leq TH \end{array}\;\right., \end{array}$$

where *η* is the random number generated through the uniform distribution probability function between [0,1]. *δ*_*d*_ represents the distance between the *d*-th dimensional value of the current global optimum position and the *d*-th dimensional value of the *i*-th bat, which is mathematically expressed as follows:

(8) $$\begin{array}{c} \delta_{d}=\left|gb_{d}-x_{i\;d}^{t-1}\right|. \end{array}$$

When *δ*_*d*_ is less than a given threshold *TH*, the *i*-th bat can fly for food at will; when the value of *δ*_*d*_ is greater than a given threshold *TH*, the *i*-th bat flies toward the current global optimal position. This strategy ensures the global search ability of the bat population to fly toward food.

In the local search process, QMBA no longer uses the random walk search strategy but decides the selection of the mutation strategy based on the mutation probability *p*_m_.

The first mutation strategy is to use the quantum computing mutation operator, which is expressed as follows:

(9) $$\begin{array}{c} x_{i\;d}^{t}=\left\{\begin{array}{c} gb_{d}^{t}+\mu \times \left|m{\text{best}}_{d}-x_{i\;d}^{t}\right|\times \;\ln\left(1/U\right),r\text{and}<0.5\\ gb_{d}^{t}-\mu \times \left|m{\text{best}}_{d}-x_{i\;d}^{t}\right|\times \;\ln\left(1/U\right),r\text{and}\geq 0.5 \end{array}\right., \end{array}$$

where both *U* and *rand* are the random numbers generated by the uniform distribution probability function between [0,1]. *μ* is an adaptive linearly decreasing weighting factor that can be expressed as follows:

(10) $$\begin{array}{c} \mu =\mu_{\text{max}}-\frac{t\left(\mu_{\text{max}}-\mu_{\text{min}}\right)}{t_{\text{max}}}. \end{array}$$

where *μ*_max_ and *μ*_min_ denote the initial and final values of *μ*, respectively.

*m*best represents the average of the current optimal position of all bats during the *t*-th iteration, i.e., the average optimal position, which can be obtained from the following equation:

(11) $$\begin{array}{c} \text{mbest}=\frac{1}{M}\left(\overset{M}{\underset{i=1}{\sum }}P_{i1}^{t},\overset{M}{\underset{i=1}{\sum }}P_{i2}^{t},\cdots ,\overset{M}{\underset{i=1}{\sum }}P_{i\;D}^{t}\right), \end{array}$$

where *P*_*i*_^*t*^ denotes the current optimal position of the *i*-th bat, *M* denotes the population size, and *D* denotes the dimension of the problem.

The second mutation strategy, which also introduces the average optimal position into the mutation operator, is expressed as follows:

(12) $$\begin{array}{c} \;x_{i}^{t}=x_{i}^{t-1}+\left(\text{mbest}-x_{i}^{t-1}\right)\phi , \end{array}$$

where *ϕ* denotes the random number generated by a uniform distribution between [0,1].

Both mutation strategies introduce the average optimal position to guide the local search, which can improve the accuracy of the search and speed up the convergence of the algorithm due to the use of statistical information of bat positions.

### 4.3. Gaussian Quantum Bat Algorithm with Direction of Mean Best Position

The QMBA algorithm improves the global search capability and accuracy of the algorithm by introducing the mechanism of the distance adaptive adjustment step, quantum computing mutation operator, and average optimal position-oriented mechanism on the basis of BA. However, the probability density functions used to generate the random numbers in QMBA are all uniformly distributed. Several works [30, 31] have shown that long-tailed distributions such as Gaussian distributions are able to perform more accurate searches in the region near the previous generation of individuals, improving the local search capability while providing larger search steps and random walk distances. Expanding the search space can improve the ability of the algorithm to jump out of the local optimum. Based on the above findings, this paper proposes the Gaussian quantum bat algorithm with direction of mean best position (GQMBA) for quantum behavior bats using a Gaussian distribution [32].

In GQMBA, random numbers are no longer generated by the uniformly distributed probability density function. To meet the requirements of the quantum computing mutation operator for random numbers in QMBA, we use the absolute value of the Gaussian distribution probability density function, in which the mean is zero and the variance is one instead (i.e., normal distribution). The one-dimensional probability density function of abs(N(0, 1)) is expressed as follows:

(13) $$\begin{array}{c} q\left(x\right)=\frac{2}{\sqrt{2\pi }}exp\left(-\frac{x^{2}}{2}\right),\;x\geq 0, \end{array}$$

GQMBA modifies the three formulas in QMBA accordingly. First, the parameter *η* in (14) is changed to be generated with a Gaussian distribution, and (7) is modified in GQMBA as follows:

(14) $$\begin{array}{c} x_{id}^{t}=\left\{\begin{array}{c} x_{id}^{t-1}+\left(gb_{d}-x_{id}^{t-1}\right)G,\;\delta_{d}>TH\\ x_{id}^{t-1}+\epsilon ,\delta_{d}\leq TH \end{array}\right., \end{array}$$

where *G* = *abs*(*N*(0,1)).

Similarly, substituting for the parameter *U* in (9), the modified quantum computing mutation operator is expressed as follows:

(15) $$\begin{array}{c} x_{id}^{t}=\left\{\begin{array}{c} gb_{d}^{t}+\mu \times \left|m{\text{best}}_{d}-x_{id}^{t}\right|\times \;\ln\left(1/G\right),r\text{and}<0.5\\ gb_{d}^{t}-\mu \times \left|m{\text{best}}_{d}-x_{id}^{t}\right|\times \;\ln\left(1/G\right),r\text{and}\geq 0.5 \end{array}\right.. \end{array}$$

Since *q*(0) = 0, *G* = *abs*(*N*(0,1)) satisfies the domain of definition of the function ln().

Finally, the random number *ϕ* in (12) is replaced with (16) as follows:

(16) $$\begin{array}{c} \;x_{i}^{t}=x_{i}^{t-1}+\left(\text{mbest}-x_{i}^{t-1}\right)G\;. \end{array}$$

**Figure 1:**
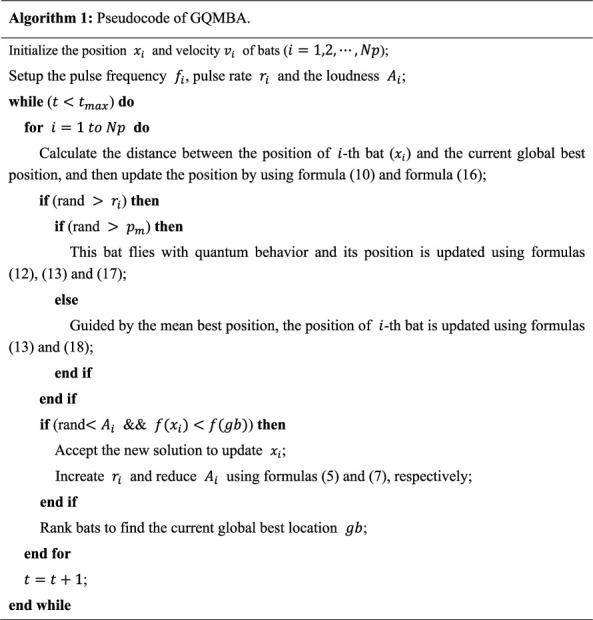
Pseudocode of GQMBA.

The pseudocode of GQMBA is given by Figure 1, in which *Np* denotes the total number of bats.

Our first significant contribution is that the Gaussian distribution is introduced in QMBA to generate random numbers. The theoretical analysis above shows that the strategy can enhance the ability of the algorithm to jump out of the local optimum and avoid premature convergence. Therefore, it is applied to the optimization problem of the cutting parameter in this paper.

### 4.4. Divide-and-Conquer Strategy for the Optimization Problem in Multipass Turnings

**Figure 2:**
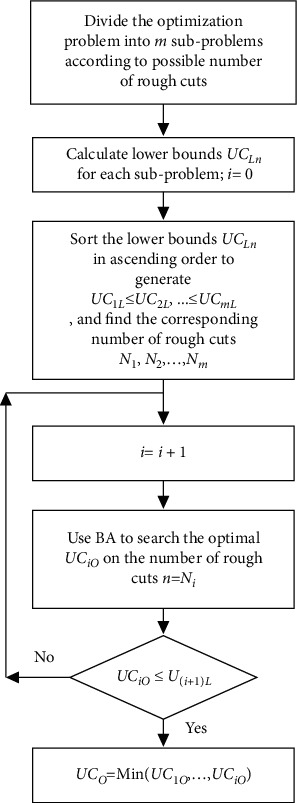
Flow chart of the divide-and-conquer strategy.

To improve the performance of the algorithm, the idea of the divide-and-conquer strategy is used to decompose the original problem into several subproblems, which can reduce the complexity of the original optimization problem. For each subproblem, the number of rough cuts is a fixed value. By conquering the subproblems one by one, the whole optimization problem can be solved. In addition, we calculate the theoretical lower bound on UC for each subproblem. Modified BA is first used to search for the optimal solution in the case of the minimum theoretical lower bound on UC, thus hopefully reducing the enumeration of the subproblems. The divide-and-conquer strategy is depicted in Figure 2 and described as follows:

1. Divide the optimization problem into *m* subproblems based on the number of possible combinations of rough cuts.
2. Calculate the theoretical lower bound on UC for each subproblem *UC*_*iL*_.
3. Sort theoretical lower bound *UC*_*iL*_ for all subproblems in ascending order. *UC*_*1L*_ ≤ *UC*_*2L*_, ..., ≤ *UC*_*mL*_ are called the first theoretical lower bound, the second theoretical lower bound ..., the *m*-th theoretical lower bound, and the corresponding numbers of rough cuts *N*_*1*_, *N*_*2*_,…, *N*_*m*_ (*N*_*i*_ is the number of rough cuts corresponding to *UC*_*iL*_) [16].
4. Starting from subproblem *i*, BA is used to solve subproblem *i*, and the optimal solution, *UC*_*iO*_, is obtained.
5. If all subproblems are enumerated or the *UC*_*iO*_ found is less than the theoretical lower bound of subsequent subproblems *UC*_(*i*  + 1)L_, the method terminates and the optimal solution is output.

### 4.5. The Framework of the Proposed Algorithm Based on GQMBA and the Divide-and-Conquer Strategy

**Figure 3:**
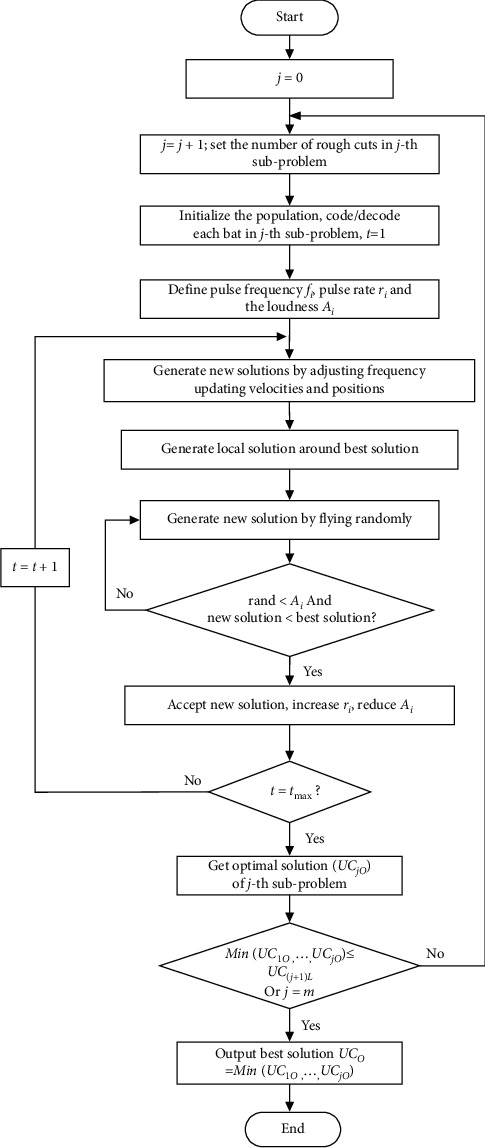
The framework of the proposed algorithm (GQMBA-DC) based on GQMBA with a divide-and-conquer strategy.

By dividing the complicated multipass turning optimization problem into simple subproblems, the optimization problem can be solved by solving these subproblems one by one. The framework of the optimization algorithm based on GQMBA and the divide-and-conquer strategy (referred to as the GQMBA-DC algorithm) is shown in Figure 3, and the main steps are as follows:

1. Divide the optimization problem into *n* subproblems based on the number of possible combinations of rough cuts.
2. Let *i* = *i* + 1; set the number of rough cuts *n* *=* *N*_*i*_*;* and start the search in the *i*-th subproblem.
3. Initialize the population, develop appropriate encoding and decoding strategies for each subproblem for GQMBA, and set the current iteration number *t* = 1.
4. Initialize the parameters and set the ultrasonic frequency *f*_*i*_, ultrasonic emission rate *r*_*i*_, and ultrasonic loudness *A*_*i*_.
5. The global search and local search are controlled by continuously adjusting the acoustic frequency and loudness to update the speed and position to generate new solutions. For the GQMBA-DC, the main body of the BA is retained, but the position update formula and the local search strategy are different. The position update formula introduces a mechanism for adaptively adjusting the step size according to the distance, while the local search strategy also introduces the average optimal position to guide the local search.
6. If *A*_*i*_ is greater than the random value *rand* and the current solution is the optimal solution, perform the next step; otherwise, return to step (5).
7. Accepting the new solution increases *r*_*i*_ and decreases *A*_*i*_ (as a bat gets closer to the target, the two values change to achieve more accurate localization).
8. Repeat the above steps until the maximum number of iterations is reached, and the GQMBA stops.
9. At present, the optimal solution of the *i*-th subproblem is obtained.
10. If *Min*(*UC*_*1O*_..., *UC*_*iO*_) ≤ *U*_*(i+1)L*_ or *i* *=* *m*, then execute the next step. Otherwise, return to step (2) to start the process of solving the next subproblem.
11. Select the minimum solution, which is the best optimal solution, from the obtained optimized solutions. Finally, output the global optimal solution *UC*_*o*_ = *Min*(*UC*_*1O*_.…, *UC*_*iO*_), and terminate the algorithm.

### 4.6. Handling of Constraints

The processing of constraints is very important for the swarm intelligence optimization algorithm; constraint processing by adding a penalty function is one of the common methods in optimization algorithms. The penalty function is a kind of constraint function. In the process of finding the optimal solution of the algorithm, the objective function is calculated by combining the penalty function, which can gradually eliminate solutions that do not satisfy the constraints and retain solutions that satisfy the constraints.

For the handling of constraints in the optimization algorithm, the bats (individuals) that violate the constraints are penalized using a penalty function to reduce the value of the objective. Different levels of penalties are imposed for different constraint violations. The more constraints that are violated, the heavier the penalty will be. Thus, by using a reasonable penalty function, the objective function value can converge to the direction of the optimal solution. The penalty function is expressed as

(17) $$\begin{array}{c} \text{penalty}\left(X\right)=\;\overset{k}{\underset{i=1}{\sum }}a_{i}+h_{i},\text{ where }a_{i}=\;\left\{\begin{array}{c} 0,\text{ satisfy constraints}\\ 1,\text{ violate constraints} \end{array}\right., \end{array}$$

where *k* is the number of constraints and *h*_*i*_ is a nondimensional constraint violation.

## 5. Simulation Experiments

During the machining processes, a cut tool is used for both roughing cuts and finishing cuts. Due to different machining conditions, the tool wear rates for rough and finish turnings are usually different. The tool life equation can be expressed as follows: *T*_*p*_ = *θT*_*r*_ + *(1* − *θ)T*_*s*_. Some studies use another tool life calculation formula: *T*_*p*_ = *T*_*r*_ *+* *T*_*s*_. Therefore, this paper separately compares the performance of the algorithms under different tool life formulations. The algorithm in this paper is implemented in the MATLAB programming language. The parameters of the BA are set as follows:

  Population size: 200
  Maximum number of iterations: 400
  Initial loudness: *A* = *u*(0, 1)
  Initial pulse emission rate: *r*_*0*_ = 0.001
  Loudness update: *α* = 0.9
  Emission rate update: *γ* = 0.9
  Threshold: *TH* = 0.005
  Mutation probability: *P*_*m*_ = 0.01

Machining examples from the literature [3–8] were used to test the performance of the optimization algorithm with the specific parameters shown in Table 1. Additionally, two different machining optimization problems with cutting depths of 6 mm and 8 mm were tested.

The algorithm was run 100 times independently on a Windows platform (CPU E3 3.5 GHz and 16 GB memory). The average value of UC was given and compared with the results obtained by previous algorithms, such as SA/PS [3], FE-GA [5], MGA [6], ACO [6], and PSO [9]. The average UC, standard deviation, number of search points, and running time for each algorithm are shown in Tables 2-5. The best results have been underlined and bolded in Tables 2-5.

Tables 2–4 show that the average UCs obtained by the proposed GQMBA-DC are smaller than those given by other algorithms. The standard deviations of the results are small, which in turn indicates that the algorithm is stable. The proposed algorithm can find the optimization results within 30 seconds for different tool life formulas and cutting depths, which shows that the proposed algorithm is an efficient algorithm. Specifically, as shown in Tables 2 and 3, the results of both cases of GQMBA-DC outperform PSO [9] when the tool life equation is *T*_*p*_ *=* *T*_*r*_ *+* *T*_*s*_. As shown in Table 4, when the tool life equation is *T*_*p*_ = *θT*_*r*_ + (*1* − *θ*)*T*_*s*_, the proposed GQMBA-DC can save 10% compared with the result given by the MGA [6]. Compared with other algorithms, such as SA/PS [3], FE-GA [5], HC [6], NM [6], ACO [6], and DP-FS [33], GQMBA-DC can further save production costs. Because the case of *d*_*t*_ = 8 cm is not covered in previous literature, only the results of our algorithm are given in Table 5. Thus, the above experimental results show that the GQMBA-DC algorithm can effectively solve the optimization problem of cutting parameters to find optimal machining parameters, which, in turn, can further reduce the production cost.

From the perspective of the optimal UC value, comparisons between the proposed GQMBA-DC and other algorithms were also conducted. The comparison of the optimal UC values in Tables 6–9 shows that the optimal UC results obtained by the GQMBA-DC are almost always smaller than the optimal results of UC obtained by other algorithms without constraint violation. The best results have been underlined and bolded in Tables 6–9. Specifically, the results found by the proposed algorithm are comparable to those achieved by HPSO [12], FPA [20], and COA [21] for the tool life equation of *T*_*p*_ = *T*_*r*_ *+* *T*_*s*_ and *d*_*t*_ = 6 mm, which is only one ten-thousandth of the difference, as shown in Table 6. The GQMBA-DC can further reduce the production cost compared with the other algorithms (i.e., HRDE [23], DERE [24], DE [24], and HRTLBO [26]), as shown in Table 6. A similar situation can also be found in Tables 7–9 in different test examples. In addition, the optimal combination of cutting parameters (*V*_*r*_, *V*_*s*_, *f*_*r*_, *f*_*s*_, *d*_*r*_, *d*_*s*_) for different cases is also given in Tables 6–9. The GQMBA-DC algorithm can find better results than the previously proposed algorithms in terms of both the average UC and the best UC. Thus, it is clear that the proposed GQMBA-DC can perform significantly better than other algorithms on solution quality in CNC turnings. Therefore, the algorithm combining the modified BA with the divide-and-conquer strategy is effective.

To overcome the different complex optimization problems in various fields, we need to carefully consider the characteristics of the specific problem and use the specific characteristics (domain knowledge) to design the optimization algorithm. In our work, for the optimization problem of machining parameters, since the machining process can be divided into different numbers of roughing cuts, we decompose the whole optimization problem of machining parameters into several simple subproblems according to the different numbers of roughing cuts. Each subproblem can be conquered individually, which greatly reduces the space of the problem solution. At the same time, to avoid enumerating all subproblems and save calculation time, we derived the theoretical lower bound on UC for each subproblem by using the characteristics of the subproblems. Then, the algorithm first searches the solution space from the subproblem with a smaller theoretical lower bound on UC. By following these steps, the algorithm can quickly find the optimal solution to the problem.

On the other hand, the performance of the combination of traditional divide-and-conquer strategy and swarm intelligence algorithm is better than the algorithms that only use traditional mathematical methods or swarm intelligence algorithms, as proven by the simulation experiments.

**Figure 4:**
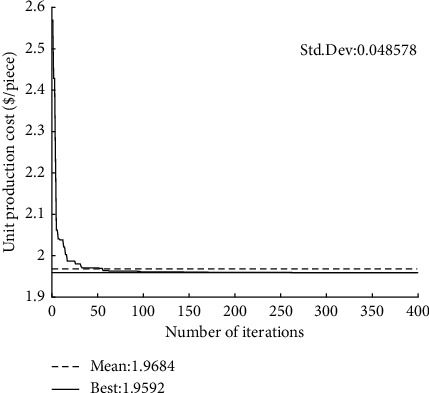
Convergence curve of the proposed GQMBA-DC (when *T*_*p*_ *=* *T*_*r*_ *+* *T*_*s*_, *d*_*t*_ = 6 mm).

**Figure 5:**
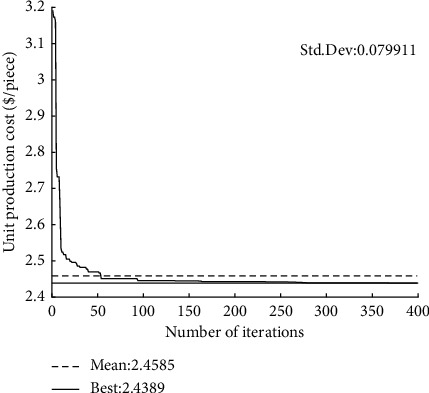
Convergence curve of the proposed GQMBA-DC (*T*_*p*_ *=* *T*_*r*_ *+* *T*_*s*_, *d*_*t*_ = 8 mm).

**Figure 6:**
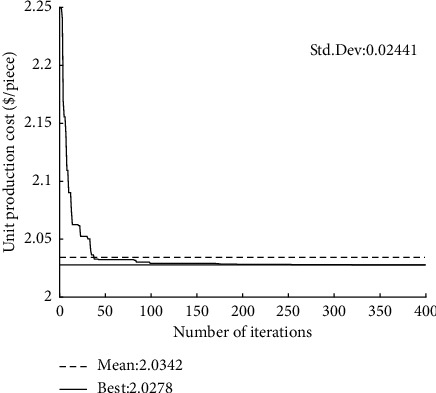
Convergence curve of the proposed GQMBA-DC (when *T*_*p*_ *=* *θT*_*r*_ *+* *(1-θ)T*_*s*_, *d*_*t*_ = 6 mm).

**Figure 7:**
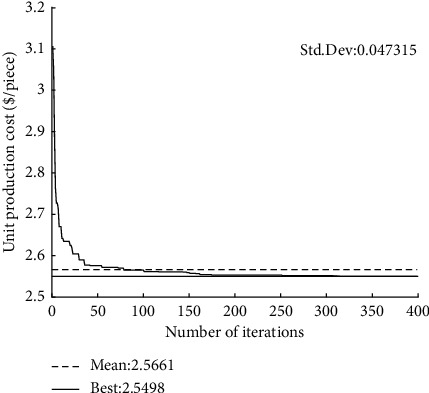
Convergence curve of the proposed GQMBA-DC (when *T*_*p*_ *=* *θT*_*r*_*+ (1-θ)T*_*s*_, *d*_*t*_ = 8 mm).

The convergence curves of GQMBA-DC for different mathematical models (tool life equation) and test cases are shown in Figures 4–7. The proposed algorithm converges to the final solution after approximately 150 generations, which indicates that the algorithm converges quickly to find satisfactory results.

## 6. Conclusions and Future Work

To solve the nonlinear optimization problem of machining parameters in CNC turnings, this paper proposes an optimization algorithm combining the bat algorithm and the divide-and-conquer strategy. First, based on the classical BA, the Gaussian quantum bat algorithm with direction of mean best position (GQMBA) is proposed by using a Gaussian distribution to generate random numbers. Second, the divide-and-conquer strategy is used to divide the complicated optimization problem into several subproblems and conquer them one by one. The simulation results show that the GQMBA-DC algorithm proposed in this paper has a stronger search capability than previous algorithms. Specifically, the proposed algorithm can find a better cutting parameter set and further reduce the production cost.

Future research can be considered from two aspects. From the algorithmic point of view, the emerging swarm intelligence algorithm can also be applied to the optimization problem, which may be able to find a better combination of machining parameters, thus reducing costs. In recent years, deep learning methods have been widely applied in various studies; deep learning methods may be considered to reconstruct mathematical models in the optimization of turning parameters [34]. On the other hand, from the perspective of new machining types, to improve the machining efficiency and quality, there are multiple tools to realize machining operations simultaneously in modern CNC turnings. Therefore, research on this type of machining optimization problem is also of great concern.

## Figures and Tables

**Table 1:** Condition parameters for turning examples.

*D* = 50 mm	*L* = 300 mm	*d*_*t*_ = 6 mm	*V*_*rL*_ = 50 m/min
*f*_*rL*_ = 0.1 mm/rev	*d*_*rL*_ = 1 mm	*V*_*rU*_ = 500 m/min	*f*_*rU*_ = 0.9 mm/rev
*d*_*rU*_ = 3 mm	*V*_*sL*_ = 50 m/min	*f*_*sL*_ = 0.1 mm/rev	*d*_*sL*_ = 1 mm
*V*_*sU*_ = 500 m/min	*f*_*sU*_ = 0.9 mm/rev	*d*_*sU*_ = 3 mm	*p* = 5
*q* = 1.75	*r* = 0.75	*μ* = 0.75	*ν* = 0.95
*η* = 0.85	*λ* = 2	*υ* = –1	*τ* = 0.4
*ϕ* = 0.2	*δ* = 0.105	*R* = 1.2 mm	*C*_0_ = 6×1011
*T*_*L*_ = 25 min	*T*_*U*_ = 45 min	*F*_*U*_ = 200 kgf	*P*_*U*_ = 5 kW
*SC* = 140	*Q*_*U*_ = 1000 °C	*SR*_*U*_ = 10 µm	*h*_1_ = 7×10−4
*h*_2_ = 0.3	*t*_*e*_ = 1.5 min/edge	*t*_*c*_ = 0.75 min/piece	*k*_*t*_ = 2.5 $/edge
*k*_0_ = 0.5 $/min	*k*_1_ = 108	*k*_2_ = 132	*k*_3_ = 1
*k*_4_ = 2.5	*k*_5_ = 1		

**Table 2:** Comparison of average UC among different algorithms (when *T*_*p*_ *=* *T*_*r*_ *+* *T*_*s*_, *d*_*t*_ = 6 mm).

Algorithm	Average UC ($)	Standard deviation	Search points (pcs)	Running time (sec)
PSO [9]	>2.2721	N/A	2,000	N/A
**GQMBA-DC**	**1.9592**	0.00005	80,000	28.4

**Table 3:** Comparison of average UC among different algorithms (when *T*_*p*_ *=* *T*_*r*_ *+* *T*_*s*_, *d*_*t*_ = 8 mm).

Algorithm	Average UC ($)	Standard deviation	Search points (pcs)	Running time (sec)
PSO [9]	>3.306	N/A	2,000	N/A
**GQMBA-DC**	**2.4393**	0.00084	80,000	30.4

**Table 4:** Comparison of average UC among different algorithms (when *T*_*p*_ *=* *θT*_*r*_*+ (1-θ)T*_*s*_, *d*_*t*_ = 6 mm).

Algorithm	Average UC ($)	Standard deviation	Search points (pcs)	Running time (sec)
SA/PS [3]	2.2959	0.01624	18,571 × 5	27.4
FE-GA [5]	2.3091	N/A	60,000	N/A
HC [6]	2.3017	N/A	100,000	N/A
NM [6]	2.2713	N/A	100,000	N/A
ACO [6]	2.2705	N/A	100,000	N/A
MGA [6]	2.2538	N/A	100,000	N/A
DP-FS [33]	2.2974	7.6 × 10^−4^	16074 × 9	19.3
**GQMBA-DC**	**2.0280**	0.00015	80,000	27.9

**Table 5:** Comparison of average UC among different algorithms (when *T*_*p*_ *=* *θT*_*r*_*+ (1-θ)T*_*s*_, *d*_*t*_ = 8 mm).

Algorithm	Average UC ($)	Standard deviation	Search points (pcs)	Running time (sec)
**GQMBA-DC**	**2.5499**	0.00043	80,000	29.2

**Table 6:** Comparison of different algorithms (when *T*_*p*_ *=* *T*_*r*_ *+* *T*_*s*_, *d*_*t*_ = 6 mm).

Algorithm	Cutting speed (m/min)		Feed rate (mm/rev)		Depth of cut (mm)		UC ($/piece)	Constraint violation
	*V*_*r*_	*V*_*s*_	*F*_*r*_	*f*_*s*_	*d*_*r*_	*d*_*s*_		
**GQMBA-DC**	123.3360	169.9697	0.5655	0.2262	3	3	**1.9592**	0
HPSO [12]	123.3424	169.9783	0.5655	0.2262	3	3	**1.9591**	0
FPA [20]	123.3431	169.9785	0.5655	0.2262	3	3	**1.9591**	0
COA [21]	123.1462	169.9876	0.5655	0.2262	3	3	**1.959**	0
GA [4]	1114.22	164.369	0.7	0.2978	2.9745	2.9863	1.7842	0.5148
ACO [7]	103.05	162.02	0.9	0.24	3	3	1.8450	0.5396
PSO [9]	106.69	155.89	0.897	0.28	2	2	2.2721	0
HRDE [23]	–	–	–	–	–	–	2.0461	–
AIA [23]	–	–	–	–	–	–	2.12	–
DERE [24]	–	–	–	–	–	–	2.046	–
ABC [24]	–	–	–	–	–	–	2.118	–
DE [24]	–	–	–	–	–	–	2.136	–
HABC [25]	–	–	–	–	–	–	2.046	–
HRTLBO [26]	–	–	–	–	–	–	2.046	–
FA [27]	98.4102	162.2882	0.82	0.2582	3	3	1.824	(24)

**Table 7:** Comparison of different algorithms (when *T*_*p*_ *=* *T*_*r*_ *+* *T*_*s*_, *d*_*t*_ = 8 mm).

Algorithm	Cutting speed (m/min)		Feed rate (mm/rev)		Depth of cut (mm)		UC ($/piece)	Constraint violation
	*V*_*r*_	*V*_*s*_	*f*_*r*_	*f*_*s*_	*d*_*r*_	*d*_*s*_		
**GQMBA-DC**	119.1460	164.2166	0.6564	0.2625	2.6673	2.6613	**2.4384**	0
HRDE [23]	–	–	–	–	–	–	2.4791	–
AIA [23]	–	–	–	–	–	–	2.51	–
DERE [24]	–	–	–	–	–	–	2.4793	–
HABC [25]	–	–	–	–	–	–	2.4790	–
ABC [24]	–	–	–	–	–	–	2.503	–
DE [24]	–	–	–	–	–	–	2.512	–

**Table 8:** Comparison of different algorithms (when *T*_*p*_ *=* *θT*_*r*_*+ (1-θ)T*_*s*_, *d*_*t*_ = 6 mm).

Algorithm	Cutting speed (m/min)		Feed rate (mm/rev)		Depth of cut (mm)		UC ($/piece)	Constraint violation
	*V*_*r*_	*V*_*s*_	*f*_*r*_	*f*_*s*_	*d*_*r*_	*d*_*s*_		
**GQMBA-DC**	109.6727	169.9756	0.5655	0.2262	3	3	**2.0278**	0
SA-PS [3]	–	–	–	–	–	–	2.3135	0.0667
HPSO [9]	109.6655	169.9796	0.5655	0.2262	3	3	2.0351	0
FPA [20]	109.6631	169.9785	0.5655	0.2262	3	3	2.0351	0
COA [21]	117.9322	123.1993	0.5655	0.2262	3	3	2.2390	0

**Table 9:** Comparison of different algorithms (when *T*_*p*_ *=* *θT*_*r*_*+ (1-θ)T*_*s*_, *d*_*t*_ = 8 mm).

Algorithm	Cutting speed (m/min)		Feed rate (mm/rev)		Depth of cut (mm)		UC ($/piece)	Constraint violation
	Vr	Vs	Fr	fs	Dr	ds		
**GQMBA-DC**	106.0251	164.2238	0.6563	0.2624	2.6670	2.6660	**2.5495**	0
SA-PS [3]	–	–	–	–	–	–	2.7411	0

## Data Availability

The data used to support the findings of this study are included in the paper.
